# Metabolic Disorders in Liver Transplant Recipients: The State of the Art

**DOI:** 10.3390/jcm13041014

**Published:** 2024-02-09

**Authors:** Filippo Gabrielli, Lucia Golfieri, Fabio Nascimbeni, Pietro Andreone, Stefano Gitto

**Affiliations:** 1 Internal and Metabolic Medicine, Department of Medical and Surgical Sciences for Children & Adults, AOU di Modena, University of Modena and Reggio Emilia, 41126 Modena, Italy; 2 Department of Surgical Sciences, University of Bologna, 40126 Bologna, Italy; 3 Clinical Psychology Unit, IRCCS Azienda Ospedaliero-Universitaria di Bologna, Policlinico di Sant’Orsola, 40138 Bologna, Italy; 4 Postgraduate School of Allergology and Clinical Immunology, University of Modena and Reggio Emilia, 41126 Modena, Italy; 5 Department of Experimental and Clinical Medicine, University of Florence, Largo Brambilla 3, 50134 Florence, Italy

**Keywords:** liver transplantation, weight gain, metabolic complications, post-transplant diabetes mellitus

## Abstract

Liver transplantation represents a chief therapeutic approach for acute liver failure, end-stage liver disease and hepatocellular carcinoma. Despite witnessing advancements in short- and medium-term survival over recent decades, attributed to refinements in surgical techniques and immunosuppressive protocols, long-term mortality remains impervious to modification. Notably, cardiovascular disease emerges as a predominant cause of mortality among liver transplant recipients. This trend is accentuated by the increasing prominence of non-alcoholic steatohepatitis-related cirrhosis as an indication for liver transplantation. Moreover, the administration of immunosuppressive agents is intricately linked to the degradation of the metabolic profile in liver transplant recipients, thereby contributing to the initiation or exacerbation of cardiovascular risk factors, such as hypertension, diabetes, and dyslipidaemia. In addition, the post-liver transplantation period is marked by a decline in lifestyle quality and a failure to acknowledge the psychological distress experienced by patients throughout the transplant process. These factors can precipitate a deterioration in the patient’s metabolic profile, exacerbated by suboptimal therapeutic compliance. This narrative review aims to comprehensively address the principal metabolic disorders intricately associated with liver transplantation.

## 1. Introduction

Exactly 61 years have elapsed since the first orthotopic liver transplantation (LT) in humans, performed by Starzl et al. At that time, short-term survival was recorded at 0%. Presently, a patient undergoing LT exhibits a 10-year survival rate ranging between 61 and 65% [1]. In the past, the primary challenges associated with the survival of LT were attributed to acute rejection or surgical complications. However, with the introduction of immunosuppressive therapy and advancements in surgical techniques, the proportion of short-term mortality attributed to these complications has diminished [2,3]. Conversely, long-term mortality has remained largely unchanged over time, with the leading causes of death in these patients encompassing graft failure, infections, metabolic and cardiovascular (CV) complications, and cancer [4].

Several studies have demonstrated that in LT, the concurrent presence of type 2 diabetes mellitus (T2DM), arterial hypertension, and the indication for liver transplantation (i.e., non-alcoholic fatty liver disease, NAFLD) can impact the overall outcome [5,6].

The issue of metabolic and cardiovascular complications in LT patients has gained considerable importance, originating both before and after LT. Specifically, concerning the pre-LT phase, the epidemic of obesity, diabetes, and metabolic syndrome (MetS) observed in recent decades has led to an increased susceptibility to cardiovascular pathologies in the general population, consequently affecting liver disease patients. Moreover, non-alcoholic steatohepatitis (NASH) is emerging as the primary cause for inclusion in the LT waiting list in the United States. It is estimated to contribute to 19.3% of the pool of LT candidates in the USA in 2021, ranking second only to alcohol-associated liver disease [7]. In certain subcategories, such as patients aged over 65 years, NASH has become the most prevalent indication for liver transplantation [8]. It has been demonstrated that hepatic fibrosis in patients with NASH serves as the most robust predictor of mortality, encompassing both liver-related and extrahepatic causes. Specifically, cardiovascular diseases emerge as the primary cause of extrahepatic mortality in these patients [9,10,11,12,13,14].

While factors such as arterial hypertension, T2DM, dyslipidemia, MetS, and obesity, often associated with NASH and heightened cardiovascular and metabolic risks, may already be present before liver transplantation, the simultaneous use of immunosuppressive medications and a more sedentary lifestyle post transplantation can further exacerbate or introduce new metabolic components [15,16,17]. For this reason, it is crucial to pay particular attention to MetS and its components in liver transplant recipients.

This review critically assesses metabolic risk factors contributing to long-term mortality in LT recipients, with a particular focus on the heightened occurrence of cardiovascular disease. This prevalence is closely associated with pre-existing conditions and correlates with cardiovascular risk factors, notably non-alcoholic steatohepatitis (NASH). Furthermore, the review explores metabolic alterations induced or exacerbated by immunosuppression, providing a comprehensive analysis of the intricate interplay between these factors in liver transplant outcomes. Additionally, it highlights the impact of these factors on lifestyle quality and psychological well-being, emphasizing the need for comprehensive care strategies to improve post-transplant outcomes.

## 2. Materials and Methods

We developed a non-systematic review using the following electronic sources: PubMed, MEDLINE, Google Scholar, Ovid, Scopus, and Web of Science. We used the following search words: “metabolic disorders”, “non-alcoholic steatohepatitis”, “metabolic-associated steatotic liver”, “liver transplant”, “diabetes”, “metabolic syndrome”, and “cardiovascular risk” alone or in combination with “outcome”, “epidemiology”, and “graft survival”. We considered all papers reporting human-related data (inclusion criteria), excluding articles with unavailable full text, not in the English language, abstracts, book chapters, and articles published before 1990 (exclusion criteria). We then examined supplementary references/articles among manuscripts considered in the first research round.

## 3. Metabolic Disorders

**Figure 1:**
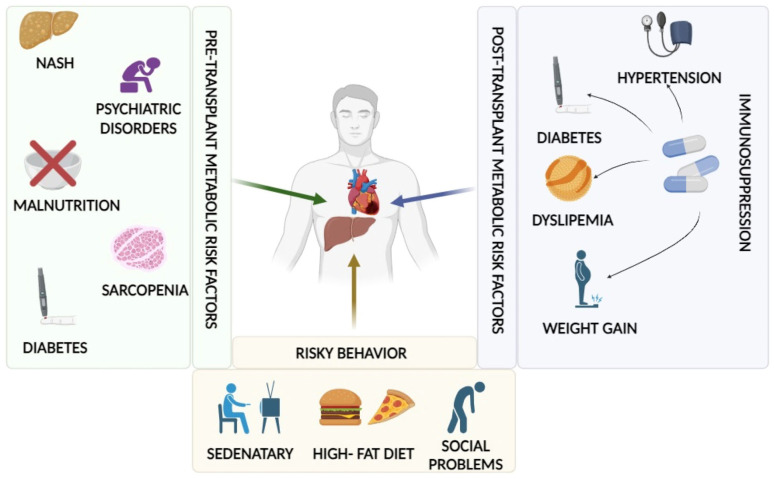
Metabolic risk factors in LT recipients. On the left are pre-liver transplant metabolic risk factors. At the bottom are depicted risky behaviors that may expose the patient to an increased risk of metabolic complications. On the right, post-transplant metabolic risk factors are listed, also related to immunosuppression regimen, which can exacerbate previous risk factors or induce new ones.

Metabolic disorders in LT recipients may either be inherited from pre-transplant conditions or arise as a consequence or exacerbation of conventional immunosuppressive regimens (corticosteroids, calcineurin inhibitors, and m-TOR inhibitors), compounded by an inappropriate lifestyle. In the following paragraphs, we will assess the contribution of major cardiovascular risk factors in patients who have undergone liver transplantation. Below (Figure 1) are summarized the metabolic risk factors that may impact the patient undergoing LT.

### 3.1. Arterial Hypertension

While arterial hypertension is one of the most prevalent cardiovascular risk factors in the general population, the incidence of post-liver transplantation hypertension is remarkably high, ranging between 50% and 80%, as reported by some studies [4,18,19]. The onset of de novo arterial hypertension post liver transplantation, as reported in a study conducted by de Oliveira Lemos et al. [20], occurred at an average of 9 ± 6.98 months. The prevalence of arterial hypertension significantly increases in liver transplant recipients, ranging from 21% to 56% [21,22,23,24]. Some of these patients may experience transient arterial hypertension that resolves in the subsequent months, allowing for the discontinuation of antihypertensive medications [25]. This is partly attributed to an increase in peripheral resistances and a reduction in vasodilatory substances following liver transplantation in patients with liver cirrhosis [25]. In addition to this underlying pathophysiological mechanism, the use of high doses of various immunosuppressive drugs to prevent acute transplant rejection further contributes. Specifically, mTOR inhibitors (mTORi) and corticosteroids are associated with the emergence of arterial hypertension in individuals previously normotensive [25,26,27]. Various liver transplantation guidelines do not establish a specific blood pressure target cutoff. However, given the heightened cardiovascular risk in these patients, the blood pressure target is typically set lower than that for the general population, often defined with a cut-off below 130/80 mmHg [28]. Typically, as a first-line therapeutic approach, calcium channel blockers are used, primarily for their vasodilatory effect on the hepatic artery [28]. In secondary consideration, selective beta-receptor blockers, angiotensin-converting enzyme inhibitors, angiotensin II receptor blockers, and loop diuretics are employed, with special attention to renal function [29,30]. The initiation of antihypertensive therapy should be preceded by lifestyle modifications, including weight loss, if necessary, increased physical activity, and a reduction in sodium intake.

### 3.2. Dyslipidemia

Dyslipidemia, defined as an imbalance in the lipid profile characterized by an increase in pro-atherogenic lipoproteins (low-density lipoprotein (LDL) cholesterol, triglycerides, apolipoprotein B) and a decrease in high-density lipoproteins (HDLs), is common among post-liver transplantation patients. Some authors report that 40–71% of post-LT patients may experience dyslipidemia, imposing the additional burden of cardiovascular risk [31,32]. The development of dyslipidemia in post-liver transplantation patients is multifactorial and can be exacerbated by the use of immunosuppressive agents [33], reduced physical activity, and weight gain [16]. The impairment of the lipid profile manifests within the initial 6 months following liver transplantation, coinciding with the administration of high doses of immunosuppressive drugs. The development of hypercholesterolemia has been observed in 13–46% of patients within 2 years of transplantation, while hypertriglyceridemia has been documented in 15–50% of patients within the same timeframe [34,35]. Steroids are associated with hypercholesterolemia as they stimulate fatty acid synthesis, induce insulin resistance, increase the synthesis of very low-density lipoprotein (VLDL), reduce the activity of lipoprotein lipase (LPL), and enhance the activity of hydroxy-methylglutaryl coenzyme A (HMG-CoA) reductase [36,37]. Calcineurin inhibitors, such as cyclosporine and tacrolimus, are associated with dyslipidemia, as an increase in LDL and a reduction in HDL have been observed, with the effects being dependent on the serum concentration of cyclosporine [38,39], resulting in a 31% increase in LDL levels. Tacrolimus appears to have a more favorable impact on the lipid profile compared to cyclosporine, leading to a lower prevalence of dyslipidemia [40,41]. mTOR inhibitors, including everolimus and sirolimus, pose an increased risk of dyslipidemia [32,42]. This is attributed to the inhibition of LPL function and a reduction in the catabolism of apoB100 and apoCIII, resulting in elevated serum levels of triglycerides, LDL, and VLDL [43,44]. The median increase in cholesterol levels during immunosuppressive therapy with everolimus was 47.4 mg/dL (95% CI 37.5–57.3) [45]. Regarding therapy with purine antimetabolites, azathioprine (AZA), and mycophenolate mofetil (MMF), there does not appear to be a clear correlation with the onset of dyslipidemia [46]. There are no specific indications from European liver transplantation guidelines regarding the management of dyslipidemia [47]. Therefore, the management of dyslipidemia is based on the patient’s cardiovascular risk stratification, as outlined in the guidelines of the European Society of Cardiology (ESC) [48]. The guidelines of the ESC recommend considering organ transplant recipients as patients at high or very high cardiovascular risk [48]. The recommended first-line drugs are statins; however, careful attention must be given to potential interactions with immunosuppressive medications, as some are metabolized through the cytochrome P450 system. Particularly, cyclosporine, metabolized through CYP3A4, may lead to serum overexposure to statins. While tacrolimus is also metabolized by CYP3A4, it appears to have fewer interactions compared to cyclosporine [48]. Therefore, the recommended statins are fluvastatin, pravastatin, pitavastatin, and rosuvastatin, as they are metabolized through other cytochromes and are less affected by potential interactions with immunosuppressive medications [49]. In patients with statin intolerance or those who have not achieved the optimal target, the introduction of ezetimibe is a viable option [48]. Ezetimibe has proven to be effective and safe in reducing cholesterol levels in liver transplant patients [50,51,52], although an increase of 2 to 12 times in serum levels has been reported in patients undergoing therapy with cyclosporine [48]. Fibrates should be used with caution as they may reduce cyclosporine levels and cause myopathy [48].

Proprotein convertase subtilisin/kexin 9 inhibitors (PCSK9i) are human monoclonal antibodies that have demonstrated up to a 50% reduction in LDL cholesterol levels [53]. PCSK9i inhibits proprotein convertase subtilisin/kexin 9, a protein involved in the recycling of LDL receptors from the hepatocyte surface. This results in an increased number of LDL receptors and consequently a reduction in cholesterol levels [53]. There are limited studies on transplant patients; however, PCSK9 inhibitors have appeared safe and effective. It is worth noting that they may lead to a reduction in serum levels of sirolimus and cyclosporine [54]. Unfortunately, their use and prescription are limited due to their high cost.

Regarding hypertriglyceridemia, lifestyle plays a significant role in triglyceride levels and is therefore the primary measure to implement in these patients [48]. If lifestyle modifications prove insufficient, statins can reduce triglyceride levels by 10–20% [55], and icosapent ethyl, up to 4 g per day, can be added [48]. In patients in primary prevention with target LDL but hypertriglyceridemia, a combination therapy of statins with fenofibrate or bezafibrate can also be considered [48].

### 3.3. Diabetes Mellitus

The prevalence of T2DM before liver transplantation is not well defined. Some authors report a prevalence ranging from 10 to 30% in patients on the LT waiting list, although this percentage may reach up to 53% in patients with NASH awaiting transplantation [56,57,58]. In patients undergoing liver transplantation, hyperglycemia is common immediately after the transplant due to the stress induced by the procedure, high doses of steroids, and, in some cases, infections [59,60]. In such cases, the recommended therapy is insulin infusion [61]. If persistent hyperglycemia (fasting glucose ≥ 126 mg/dL or random glucose ≥ 200 mg/dL) is observed 45 days after transplantation, the diagnosis of post-transplant diabetes mellitus (PTDM) can be made [62]. This condition can be found in up to 40% of LT recipients [63,64], and it is associated with a decline in the quality of life due not only to the intrinsic complications of diabetes but also to the potential interaction between antidiabetic medications and immunosuppressive drugs [65]. Regarding post-transplant outcomes for patients with PTDM, several studies have demonstrated an increased mortality, particularly cardiovascular mortality, in PTDM patients compared to those without diabetes mellitus [4,66]. However, in multivariate analysis, the only independent predictor of cardiovascular mortality was the presence of pre-LT T2DM, being associated with approximately 10% of the mortality [64,65]. Risk factors for the development of PTDM are associated with male gender, the reason for liver transplantation, particularly cirrhosis due to NASH, HCV, and alcohol, overweight/obesity, and immunosuppression [67,68]. Among the immunosuppressive agents associated with hyperglycemia are corticosteroids, which induce both hepatic and peripheral insulin resistance and result in a reduction in insulin secretion, calcineurin inhibitors, which are directly toxic to pancreatic beta cells, and tacrolimus, which is associated with a higher incidence of PTDM [69,70,71].

The treatment of T2DM LT recipients does not differ from the treatment of a patient with T2DM, both in nutritional and pharmacological approaches. The target glycated hemoglobin (HbA1c) level to achieve should be below 7% (53 mmol/mol) in patients where the risk of hypoglycemia can be avoided or 8% (64 mmol/mol) in patients where the risks of therapy may outweigh the benefits. [72]. A conservative approach is recommended until the patient has stabilized post LT [65]. The major challenges in initiating antidiabetic therapy in these patients are primarily due to the modification of immunosuppressive therapy, pharmacological interactions, and a deterioration of the patient’s lifestyle post LT [16]. Metformin is the first-line drug, but it must be used cautiously in patients with renal insufficiency as it has the potential to trigger lactic acidosis. Additionally, in all patients, it can cause diarrhea, leading to the reduced absorption of immunosuppressive agents due to increased intestinal motility. Moreover, metformin has demonstrated an anticarcinogenic effect, particularly in reducing the risk of HCC development in patients under metformin therapy [73]. The second-line therapeutic options include glucagon-like peptide-1 receptor agonists (GLP1-RAs) and sodium-glucose cotransporter-2 inhibitors (SGLT-2i). GLP1-RAs have not been extensively studied in LT recipients, although no pharmacological interactions are expected between these agents and immunosuppressive drugs. It should be noted that one of the effects of GLP1-RAs is delayed gastric emptying, which may slow the absorption of calcineurin inhibitors and mycophenolate [74]. For SGLT-2 inhibitors, there are small studies on PTDM in patients with solid organ transplants (SOTs) suggesting their efficacy and safety [75], and a review including 2500 SOT patients demonstrated a reduction in HbA1c and weight in the short term [76]. However, attention should be paid to the potential development of urinary tract infections in these patients, and their use may not always be suitable in cases of renal insufficiency. The third-line therapeutic options include dipeptidyl peptidase-4 inhibitors (DPP-4i), drugs capable of prolonging the half-life of endogenous incretins, resulting in a hypoglycemic effect [77]. The evidence for the use of DPP-4 inhibitors in PTDM mainly comes from patients undergoing kidney transplantation. Linagliptin has been shown to provide better HbA1c reduction with minimal variation in cyclosporine serum levels, while sitagliptin demonstrated a greater reduction in weight [78,79]. In patients for whom these therapies prove ineffective or are contraindicated, the last-line therapeutic option is insulin. Finally, and no less importantly, these patients should be periodically evaluated for micro- and macrovascular complications of diabetes, with particular attention to the development of foot infections.

### 3.4. Metabolic Syndrome

MetS encompasses a cluster of pathological conditions predisposing individuals to an elevated cardiovascular risk, the development of T2DM, the onset of obstructive sleep apnea syndrome, and the initiation or progression of NASH [80]. The association of at least three of the following pathological conditions defines MetS: elevated waist circumference (≥88 cm in women and ≥102 cm in men), elevated triglycerides (≥150 mg/dL), reduced high-density lipoprotein cholesterol (HDL-C) (<40 mg/dL in men and <50 mg/dL in women), elevated blood pressure (≥130 mmHg systolic blood pressure or ≥85 mmHg diastolic pressure or drug treatment for arterial hypertension), and elevated fasting glucose (≥100 mg/dL or drug treatment for elevated glucose) [81]. The prevalence of MetS has increased over the years, both in the general adult population [82] and in specific subpopulations, such as patients undergoing LT [83]. Approximately half of the patients undergoing LT develop MetS within 3 years post LT [83], and the mortality rate in post-LT patients with MetS is four times higher than in those without MetS [84]. The development of MetS in these patients is attributed to factors such as pre-LT weight, age, the presence of diabetes, and triglyceride levels. However, it can also be exacerbated by the use of immunosuppressive medications [85]. The longer post-LT survival experienced due to improved surgical techniques, immunosuppressive therapies, and the increasing number of transplants related to cirrhosis from NASH necessitates the early recognition, prevention, and aggressive treatment of factors contributing to metabolic syndrome in LT recipients [8,80].

### 3.5. De Novo and Recurrent MASL

Metabolic dysfunction-associated steatotic liver (MASL), formerly known as non-alcoholic fatty liver disease (NAFLD), is a condition characterized by the presence of hepatic steatosis associated with either obesity/overweight, the presence of type 2 diabetes mellitus (T2DM) or prediabetes, and evidence of metabolic dysregulation [86]. The condition affects approximately 25% of the general population [87], and as mentioned earlier, cirrhosis related to it is becoming the leading cause of liver transplantation [8]. The development of de novo MASL or recurrent graft steatosis is relatively common in LT recipients [88]. In detail, after 5 years from LT, recurrent graft steatosis and NASH may be present in approximately 80% and 60% of patients transplanted for NASH cirrhosis, respectively. Some authors argue that the recurrence of the disease is essentially inevitable [89]. In a recent meta-analysis, it was reported that the development of recurrent graft steatosis is five times more likely than de novo MASL [OR: 5.38 (2.69–10.76)]. These conditions are predisposed by factors such as obesity, diabetes, and metabolic syndrome [90]. The prevalence of NASH is 29% in patients undergoing LT, with the strongest risk factor being the presence of pre-LT MASL [90]. Among immunosuppressive drugs, only sirolimus has been associated with an increased risk of post-LT hepatic steatosis [OR: 1.68 (1.07–2.64)] [90]. Recurrent MASL appears to be a more severe and aggressive form of the disease compared to de novo MASL, leading to a faster progression of fibrosis into cirrhosis within 5 years [91,92]. Currently, there is no specific therapy for these conditions. However, exercise programs and a diet tailored in consultation with specialists, along with the management of factors predisposing to metabolic syndrome, should be pursued.

## 4. Post-Transplant Lifestyle

Patients on the waiting list for a LT often experience impaired well-being due to conditions such as sarcopenia, protein–calorie malnutrition, reduced physical activity, and frequent muscle fatigue, particularly in the context of cirrhosis and HCC [93]. Despite the immediate normalization of liver function after LT, the recovery of physical function often takes longer [94]. Additionally, as mentioned earlier, post-LT issues such as hypertension, obesity [95], diabetes, MASL, and dyslipidemia can develop due to immunosuppressive therapies, potentially increasing the cardiovascular risk in these patients and further impacting their quality of life.

The impact of physical activity on reducing cardiovascular risk and the risk of T2DM, obesity, and mortality is well established in the general population [96], although it is not yet clearly determined in the subpopulation of patients undergoing LT [97]. Approximately 25% of post-LT patients engage in inadequate physical activity [16], while combined exercise and dietary counselling interventions have been associated with increased VO2peak, self-reported general health, and maximum overload [94,98]. At present, there is no unanimous consensus on the type and amount of suitable physical activity for post-LT patients. Therefore, the approach recommended by the World Health Organization (WHO) to engage in at least 150 min per week of moderate-to-vigorous physical activity, coupled with 15–20 min of resistance exercises twice weekly, might be reasonable [99]. Based on the results of a recent meta-analysis, however, further RCTs with blinded outcome assessments are needed to determine the actual effectiveness of structured physical activity in LT recipients [100].

Another important aspect to consider in LT patients is the role of nutrition. Both managing malnutrition in patients on the waiting list [101] and weight gain with worsening metabolic indices induced by immunosuppressive therapy in transplant recipients are challenging. In the post-LT period, the body’s energy and protein requirements are increased in the first 4 weeks [102]. Therefore, transplant patients should receive approximately 1.5 g/kg of proteins and 30 Kcal/kg per day [101,103]. The patient’s fluid and electrolyte status must be constantly monitored since there is a risk of electrolyte imbalances and fluid overload induced by both the therapies implemented and the risk of refeeding syndrome [104]. After the first month post LT, there is a noticeable weight increase that reaches full recovery within a year and, unfortunately, continues in the subsequent years [105,106]. Despite the weight gain, there is no recovery of muscle mass, and therefore, the additional fat mass becomes a pivotal element in the genesis of various factors of the metabolic syndrome, negatively impacting outcomes such as survival [1]. Therefore, every effort to reduce the risk of increased fat mass should be implemented, starting with educating the patient to follow a balanced diet with limited fat intake and adequate protein intake in accordance with a multidisciplinary team that includes a nutritionist [107]. Regarding diet, the Mediterranean diet has proven to be a nutritional approach capable of reducing cardiovascular risk even in liver transplant recipients [108,109]. However, a moderate-to-low adherence to the Mediterranean diet has been observed in liver transplant recipients, highlighting the importance of educating patients in the early post-liver transplant phases about the need to follow an appropriate dietary regimen [110]. Furthermore, regarding the impact of immunosuppressive agents, particular attention must be paid to corticosteroids due to their orexigenic, catabolic, and adipose tissue deposition capacities, as well as to calcineurin inhibitors, as they are independent predictors of weight gain [107]. The table (Table 1) below outlines therapeutic approaches and lifestyle modifications for metabolic risk factors in liver transplant recipients.

## 5. Innovative Approaches

End-stage liver disease, along with the wait for liver transplantation, the surgical procedure, and the subsequent post-LT follow-up with the constant risk of rejection, infection, and complications, expose the patient to various psychological distress. This can adversely affect the patient’s quality of life in all phases of the transplantation process [111,112]; indeed, a depression rate of 15% has been observed in patients on the LT waiting list [113]. Psychological and psychiatric assessments of candidates for liver transplantation can aid in the early identification of patients at an increased risk of post-LT mortality. This includes patients who have resumed alcohol use [114], those experiencing depression [115], or individuals with potential lower adherence to therapy [116]. Therefore, it is crucial to have psychologists and psychiatrists as part of the liver transplantation team to enhance the quality of life and survival prospects for LT patients [116]. Moreover, an improvement in depressive symptoms, physical activity, and social adaptations [117] has been observed in patients undergoing post-LT group psychotherapy [118]. Additionally, there is a reduction in relapses of alcohol abuse in patients with alcohol dependence who participated in group therapy before liver transplantation [119].

Furthermore, several studies have demonstrated the importance of social support in achieving better liver transplantation survival outcomes and adherence to the therapeutic regimen [120,121,122,123,124].

## 6. Conclusions

LT represents a pivotal moment in the life of a patient with liver disease. Despite advancements in surgical techniques and immunosuppression protocols, long-term mortality and patient quality of life may be negatively affected by metabolic complications arising from immunosuppressive regimens, lifestyle deterioration, or psychosocial system fragility.

Metabolic disorders in patients undergoing LT (especially for those with pre-LT for MASL) are the main challenge for transplant specialists due to the wide impact on the long-term outcome.

It is mandatory that early after LT, LT recipients and their caregivers can be accurately informed and educated to avoid uncontrolled weight gain.

Supervised diet and structured exercise programs should be recommended and implemented for all transplant recipients for both the prevention and treatment of metabolic disorders. Furthermore, a personalized approach in the use of immunosuppressive drugs is a chief point to balance the risk of rejection and metabolic risks.

A correct lifestyle approach and a wide psychosocial support can help LT recipients to achieve a better quality of life and a lower morbidity and mortality.

The transplant community should improve its effort to support patients with dedicated personal treatments and structures that can increase the capacity of the prevention and treatment of post-LT metabolic disorders.

## Figures and Tables

**Table 1:** Therapeutic and non-pharmacological approaches for metabolic risk factors in liver transplant recipients.

METABOLIC DISORDER	PHARMACOLOGICAL APPROACHES	LIFESTYLE CHANGES	REF.
ARTERIAL HYPERTENSION	Calcium channel blockers		[28,29,30]
	Selective beta-receptor blockers	Weight loss if necessary	
	Angiotensin-converting enzyme inhibitors	Increased physical activity	
	Angiotensin II receptor blockers	Reduction in sodium intake	
	Loop diuretics		
DYSLIPIDEMIA	Statin		[48,49,50,51,52,54,55]
	Ezetimibe	Weight loss if necessary	
	Fibrates	Increased physical activity	
	iPCSK9	Balanced diet	
	Icosapent ethyl		
T2 DIABETES MELLITUS	Biguanides		[72,73,75,76,78,79]
	GLP1-RAs	Weight loss if necessary	
	SGLT-2i	Increased physical activity	
	DPP-4i	Hypoglycemic diet	
	Insulin		
MASL	Addressing the altered metabolic factor	Weight loss if necessary	[99]
	No approved medications	Increased physical activity	

Diabetes mellitus = type 2 diabetes mellitus, iPCSK9 = proprotein convertase subtilisin/kexin 9 inhibitors, GLP1-RAs = glucagon-like peptide-1 receptor agonists, SGLT-2i = sodium-glucose cotransporter-2 inhibitors, DPP-4i = dipeptidyl peptidase-4 inhibitors, MASL = metabolic dysfunction-associated steatotic liver.

## Data Availability

The data presented in this study are available on request from the corresponding author.
